# Knowledge, attitudes, and practices on antimicrobial use and antimicrobial resistance among poultry practitioner veterinarians

**DOI:** 10.3389/fvets.2024.1349088

**Published:** 2024-04-09

**Authors:** Manoj Kumar Shahi, Saharuetai Jeamsripong

**Affiliations:** ^1^Nepal Veterinary Council, Kathmandu, Nepal; ^2^Research Unit in Microbial Food Safety and Antimicrobial Resistance, Department of Veterinary Public Health, Faculty of Veterinary Science, Chulalongkorn University, Bangkok, Thailand

**Keywords:** antimicrobial resistance, antimicrobial use, KAP, Nepal, poultry practitioner veterinarians

## Abstract

**Background:**

Antimicrobial resistance (AMR) poses a serious global threat to human and animal health. In the context of antimicrobial usage (AMU) in livestock production, veterinarians are key stakeholders. However, there is a lack of comprehensive situational analysis regarding the current Knowledge, Attitudes, and Practices (KAP) among veterinarians concerning AMU and AMR in poultry production in Nepal.

**Methods:**

The primary objective of this study was to evaluate the situation of AMU and KAP regarding AMU and AMR of among poultry practitioner veterinarians in Nepal. A total of 327 respondents from 56 districts across seven provinces participated. Demographic information and AMU situation were collected and analyzed using descriptive statistics, and factors affecting KAP on AMU and AMR were performed using logistic regression analysis.

**Results:**

Nearly half of veterinarians (49.2%) were from Bagmati, followed by Lumbini (16.5%) and Gandaki (8.9%) provinces. Most of the respondents (85.0%) identified themselves as male with a mean age of 31.9 ± 7.8 years, with a range of 24–74 years. A large proportion of veterinarians held a master’s degree (43.8%). Regarding reasons for AMR, 51.1% of them attributed it to the irrational use of antimicrobials. Other identified reasons for AMR, including over-the-counter sales (27.8%), low-dose administration (12.3%), and low-quality antimicrobials (6.7%). Based on antibiotic prescription rates, most veterinarians (50.8%) prescribed antimicrobials at a rate of 20–40%, while 25.9% prescribed at a rate of less than 20.0 and 18.9% at a rate of 40–60%. Approximately 89.0% of veterinarians agreed that vaccination could reduce the use of antimicrobials in poultry, and 75.6% preferred narrow-spectrum antimicrobials than broad-spectrum antimicrobials. A combination of broad-spectrum antimicrobials such as colistin with amoxicillin, gentamicin, tylosin, and tetracycline was commonly used in poultry production.

**Discussion:**

In logistic regression analysis, it was observed that veterinarians aged 45–60 years demonstrated significantly higher levels of knowledge concerning AMU and AMR (*p* = 0.02) compared to those in the 24–30 age group. This study indicates that the need for robust regulatory mechanisms in veterinary drug administration and increased awareness among veterinarians to address the AMR issue livestock production.

## 1 Introduction

Antimicrobial resistance (AMR) poses a worldwide threat from the perspective of One Health. The improper use of antimicrobial agents in both humans and animals stands as a critical factor contributing to the emergence and dissemination of drug-resistant pathogens (1). The emergence of AMR in livestock can be transmitted to humans through the food production chain (2). Globally, AMR is responsible for 700,000 annual human fatalities. It is projected that by 2050, the economic toll of AMR infections could reach 100 trillion US dollars, potentially resulting in 10 million human deaths if urgent action is not taken (3). The burden of the AMR issue is significantly greater in low and middle-income countries compared to more developed countries (4). Therefore, increasing awareness of antimicrobial use (AMU) among multiple stakeholders involved in livestock production to effectively manage and prevent AMR.

The purposes of AMU in poultry include the treatment of bacterial infections, promotion of animal growth, and control and prevention of bacterial diseases. However, the practice of using antimicrobials as growth promoters in animal feed has been banned in livestock production in different countries, including Sweden, Europe, and the United States (5, 6). Furthermore, Nepal has banned the use of AMU for growth promoters since 2017 (7, 8). The benefits of using antimicrobials in poultry have been shown to decrease disease incidence, morbidity, and mortality rates, improve animal health, and increase productivity, resulting in higher economic returns (9). However, the misuse of antimicrobials can develop conditions for the proliferation of AMR bacteria, leading to the transfer of resistance traits. The dissemination of multidrug resistance among commensal and pathogenic bacteria continues to reduce the efficacy of available antimicrobials, which were highly impacted on public health and socioeconomic aspects.

The Ministry of Health and Population in Nepal has endorsed the National Antibiotic Containment Action Plan from 2016 and the National Antibiotic Treatment Guideline from 2014 to regulate the judicious use of antimicrobials (10). However, the Drug Act of 1978 in Nepal lacks specific provisions for regulating veterinary drugs. Therefore, there is a need for a legal and efficient framework for managing AMU in livestock within Nepal. The lack of awareness among veterinarians regarding the appropriate use of antimicrobials can exacerbate the issue of AMR. The responsible use of antimicrobials is related to knowledge, attitudes, and practices (KAP). KAP surveys are a common tool for researching health-seeking behavior and serve to collect information regarding what a specific target group knows, believes, and does concerning a particular topic (11).

In the poultry industry, several factors such as self-prescription by farmers, unauthorized usage, and a lack of regulatory oversight are key drivers of the emergence of AMR. To effectively address the AMR, knowledge of AMU and AMR in animals and their impact on public health is vital to minimize the indiscriminate use of antimicrobials in poultry. Moreover, there is a need for more information regarding the economic and livestock health consequences of AMR in developing countries (12). As part of the solution within veterinary services, it is imperative that veterinarians receive proper training and are subject to supervision by authorized veterinary statutory bodies (13). Understanding how antimicrobials are used by veterinarians is the first crucial step for implementing other strategies to prevent and control AMR, since KAP of veterinarians regarding AMR significantly influence the AMU on livestock farms. Therefore, this study conducts a situational analysis of AMU and KAP among veterinarians regarding AMU and AMR. The result of this study is necessary to provide policymakers to address AMR in the country effectively.

## 2 Methodology

A cross-sectional questionnaire survey was carried out to assess KAP related to AMU and AMR among veterinarians working with broiler poultry. The questionnaires covered a range of topics, including demographic information, as well as KAP associated with AMU and AMR. The questionnaire related to demographic information of participants included of age, gender, educational background, work experience, province, and ecozone. Most of the questions were multiple-choice questions.

### 2.1 Situation of AMU

Questions related to the situation analysis of AMU were interviewed. Questions regarding the prudent use of antimicrobials such as the type and frequency of AMU were directed toward poultry practitioner veterinarians.

### 2.2 KAP questionnaire

The KAP questionnaire is structured into three sections, addressing knowledge, attitudes, and practices. The knowledge section contains questions concerning AMU in food-producing animals, antimicrobial residues, the consequences of improper usage, public health implications, and government policies and regulations related to AMU and AMR. For attitude questions, this section was related to the safety of AMU, AMR issue, strategies to combat AMR, withdrawal periods for antimicrobials, etc. In the practice section, respondents are questioned about their actual behaviors and practices regarding AMU, such as the purpose of AMU, on-demand prescription patterns, the use of single or combined antimicrobials, frequency of AMU, methods for calculating doses, adherence to national guidelines, and participation in ongoing education or training programs related to AMU and AMR.

### 2.3 Validation of the questionnaire

The questionnaire was validated using pretest and expert evaluation. Before the actual survey, a pretest was conducted with a sample of five veterinarians who tested the questionnaires. Additionally, the questionnaires were validated by three experts, including one of each practitioner, scientist, and epidemiologist using the Item-Objective Congruence (IOC) index, considering the study objectives. The Content Validity Index (CVI) was used to examine the agreement between different sections of the questionnaire and the intended measurement objectives. Experts assessed the relevance and clarity of each questionnaire section. The CVI score ranges between 0 and 1, with higher values indicating stronger content validity. The experts used a three-point scale to rate the consistency and congruence of all questions. The experts had to choose one of the following alternatives to assign a mark as (1) items not relevant or clear; (2) items somewhat relevant or clear; or (3) items highly relevant or clear. The IOC index of this study was 0.8. This validation process ensures that the questionnaire accurately measures the intended objectives and maintains high content validity.

### 2.4 Study area

**Figure 1:**
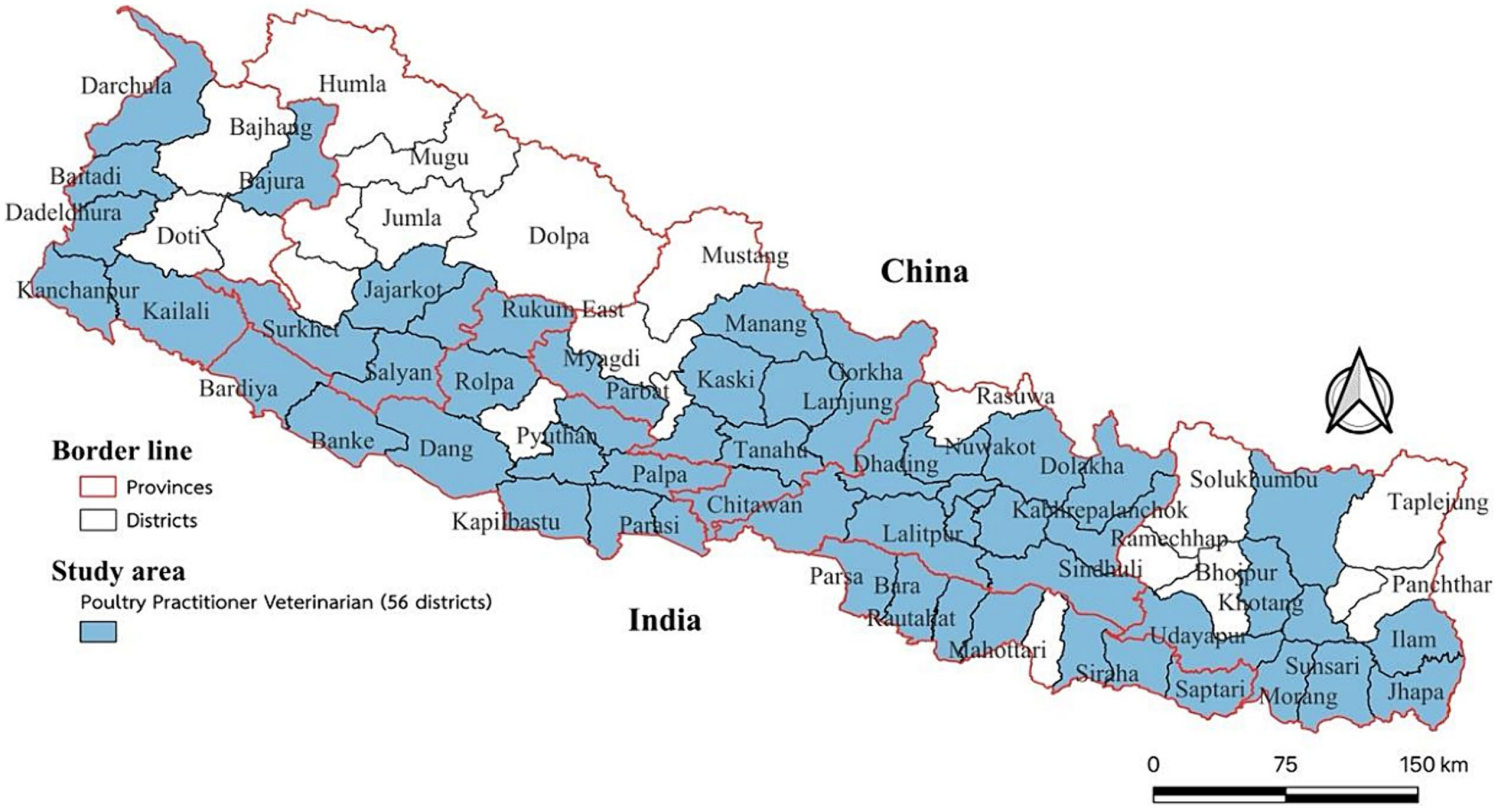
Geographical distribution of study area.

The questionnaire was distributed to all 481 poultry practitioner veterinarians across Nepal using an online survey. A total of 327 respondents met the criteria were collected from seven provinces (56 districts): Sudurpaschim province (Baitadi, Bajura, Dadeldhura, Darchula, Kailali, and Kanchanpur); Karnali province (Jajarkot, Rukum West, Salyan, and Surkhet); Lumbini province (Banke, Bardia, Dang, Gulmi, Kapilvastu, Palpa, Rolpa, Rukum East, Nawalparasi West, and Rupandehi); Gandaki province (Nawalparasi East, Arghakhanchi, Baglung, Gorkha, Kaski, Lamjung, Manang, Syangja, and Tanahun); Bagmati province (Bhaktapur, Chitwan, Dhading, Dolakha, Kathmandu, Kavrepalanchok, Lalitpur, Makawanpur, Nuwakot, Ramechhap, Sindhuli, and Sindhupalchok); Madhesh province (Bara, Dhanusha, Parsa, Rautahat, Saptari, Sarlahi, and Siraha); and Koshi province (Bhojpur, Dhankuta, Ilam, Jhapa, Morang, Sankhuwasabha, Sunsari, and Udayapur) (Figure 1).

### 2.5 Study population and sample size

The study population comprised all 1,622 registered veterinarians, with an estimated 481 of them assumed to work as poultry practitioners in Nepal (14). For this study, the respondents were chosen from the pool of veterinarians providing veterinary services to poultry farms, which includes activities such as diagnosis, treatment, and vaccination. To determine the sample size, a 95% confidence interval, and the estimated response rate of participants of 80% with a desired precision of 5% was used. The required sample size for this study was determined to be at least 245 veterinarians.

### 2.6 Questionnaire survey and data collection

The questionnaire survey for veterinarians was conducted using Google Form (Google LLC, Mountain View, CA, United States). The questionnaire was made available in both Nepali and English languages. The questionnaire link was distributed to veterinarians through various channels, including email and social media platforms such as Facebook Messenger, WhatsApp, and Viber. The sampling frame of the veterinarian were obtained from the Nepal Veterinary Council. A total of 35 questions were administrated covering Knowledge (10), Attitude (8), and Practice (15). The remaining 12 questions were related to demographic information (8) and current situation regarding AMU (4).

### 2.7 Statistical analysis

The data acquired through the questionnaire survey underwent a comprehensive analysis that included cross-checking, tabulation, cleaning, processing, and verification, using Microsoft Excel 365. The overall scores for KAP were analyzed based on the number of correct answers provided by the veterinarians. If the combination of KAP score among the respondents was equal to or below 80%, it was classified as “Unsatisfactory.” Conversely, a score exceeding 80% was considered indicative of a “Satisfactory” level of KAP. The distribution of respondents was mapped using QGIS 3.4 (Free Software Foundation, Boston, United States). Descriptive analysis was conducted to describe AMU situation and KAP among veterinarians. Binary logistic regression analyses were used to determine associations between variables related to KAP with the outcome as AMU and AMR. Independent variables with a *p*-value less than 0.1 in the univariate analysis were selected for the multivariable analysis. Statistically significant results in the univariate logistic regression analysis were defined by a *p*-value <0.05 in the final multivariable analysis. Statistical analysis was performed using STATA/SE 14 (StataCorp., College Station, TX, United States).

### 2.8 Ethical statement

Ethical approval was granted from the Nepal National Health Research Council (Reference No. 3029). Prior to the questionnaire interviews, all participants either provided written consent or accepted it in an electronic format. To safeguard participant privacy, all data collected for the study underwent anonymization.

## 3 Results

### 3.1 Demographic characteristics of veterinarians

In this study, questionnaire was distributed to 481 veterinarians of 56 districts of Nepal. This study obtained a 68.0% response rate, leading to the inclusion of 327 participants. There was a nearly equal distribution between terai (50.5%) and hill regions (45.9%) (Table 1). Approximately half of the veterinarians (49.2%) came from Bagmati province, followed by Lumbini (16.5%) and Koshi (9.2%) provinces. Most participants (85.0%) identified themselves as male. The average age was 31.9 ± 7.8 years, ranging from 24 to 74 years. The largest proportion (55.4%) of veterinarians fell within the 24–30 age group, followed by the 31–60 age group (38.2%). Most of the veterinarians (53.8 and 43.8%) had bachelor-and master-level education. Regarding their occupations, participants reported owning private businesses (41.6%), followed by employment in government service (29.1%), academia and research (22.6%).

**Table 1:** Demographic distribution of the participants (*n* = 327).

Variables	*N* (%)
Ecozone	
Terai	165 (50.5)
Hill	150 (45.9)
Mountain	12 (3.7)
Province	
Bagmati	161 (49.2)
Lumbini	54 (16.5)
Koshi	30 (9.2)
Gandaki	29 (8.9)
Madhesh	27 (8.3)
Karnali	14 (4.3)
Sudurpaschim	12 (3.7)
Gender	
Male	278 (85.0)
Female	49 (15.0)
Age group (years)	
24–30	181 (55.4)
31–45	125 (38.2)
46–60	17 (5.2)
>60	4 (1.2)
Educational level	
Bachelor	176 (53.8)
Master	143 (43.8)
Ph.D.	8 (2.4)
Type of primary job	
Private business	136 (41.6)
Government service	95 (29.1)
Academia and research	74 (22.6)
Non-government organization	22 (6.7)

### 3.2 AMU situation

Approximately, 34.4% of them emphasized the importance of using appropriate treatment guidelines, 19.7% highlighted the need for improved biosecurity and hygiene, 17.1% believed that increased education is mandatory, and 15.7% thought that controlling antimicrobial sales could help mitigate the impact of AMR (Table 2). The majority (51.1%) attributed the irrational use of antimicrobials as the primary cause of AMR. This study also identified other contributing factors to AMR, including over-the-counter sales (27.8%), low-dose administration (12.3%), and low-quality antimicrobials (6.7%). Regarding the proportion of antimicrobial prescribed, most veterinarians (50.8%) prescribed antimicrobials for 20–40% of their prescriptions, while 25.9% of them prescribed antimicrobials for less than 20.0%. Only 4.3% of veterinarians prescribed antimicrobials for more than 60% of their cases (Table 2).

**Table 2:** Situation analysis of AMU and AMR.

Variable	*N* (%)
Important strategies to combat AMR (*n* = 625)^*^	
Use of appropriate treatment guideline	215 (34.4)
Improve biosecurity and hygiene of farm	123 (19.7)
Educational campaigns	107 (17.1)
Control of antimicrobial sells	98 (15.7)
Vaccination campaigns	54 (8.6)
Reduce AMU in animal	28 (4.5)
Primary reason for AMR (*n* = 569)^*^	
Irrational use	291 (51.1)
Over-the-counter sell	158 (27.8)
Low dose administration	70 (12.3)
Low-quality antimicrobials	38 (6.7)
Other reasons	12 (2.1)
Proportion of antimicrobials in prescription (*n* = 327)	
<20%	85 (25.9)
20–40%	166 (50.8)
40–60%	62 (18.9)
>60%	14 (4.3)

^*^Multiple answers allowed.

The prescription frequency of antimicrobials in broiler poultry was categorized based on class and type of antimicrobials (Table 3). Thirteen different types of antimicrobial classes were prescribed. Most prescribed antimicrobial classes were quinolones (27.4%), tetracycline (21.7%), aminoglycosides (15.1%), macrolides (7.7%), glycopeptides (6.5%), and penicillin (6.2%), respectively. Among the specific antimicrobials, the most frequently used were enrofloxacin (12.1%), doxycycline (10.8%), tetracycline (9.3%), ciprofloxacin (7.8%), neomycin (7.7%), levofloxacin (6.8%), gentamycin (6.7%), tylosin tartrate (6.6%), colistin sulfate (6.2%), amoxicillin (5.9%), and sulfamethoxazole (4.4%).

**Table 3:** Antimicrobial classification in the poultry industry.

Antimicrobial classification	*N* (%)
Quinolones	
Enrofloxacin	88 (12.1)
Ciprofloxacin	57 (7.8)
Levofloxacin	50 (6.8)
Flumequine	5 (0.7)
Tetracyclines	
Doxycycline	79 (10.8)
Tetracycline	68 (9.3)
Chlortetracycline	12 (1.6)
Aminoglycosides	
Neomycin	56 (7.7)
Gentamycin	49 (6.7)
Amikacin	5 (0.7)
Macrolides	
Tylosin tartrate	48 (6.6)
Azithromycin	6 (0.8)
Erythromycin	2 (0.3)
Glycopeptides	
Colistin sulfate	45 (6.2)
Bacitracin	2 (0.3)
Penicillins	
Amoxicillin	43 (5.9)
Cloxacillin	2 (0.3)
Sulfonamides	
Sulfamethoxazole	32 (4.4)
Sulfadiazine	9 (1.2)
Cephalosporins	
Cephalosporin^**^	16 (2.2)
Cephalexin	10 (1.4)
Ceftiofur	4 (0.5)
Diaminopyrimidines	
Trimethoprim	13 (1.8)
Phenicols	
Florfenicol	11 (1.5)
Nitrofurans	
Furaltadone	9 (1.2)
Chloramphenicol	5 (0.7)
Lincosamides	
Lincomycin	4 (0.5)

^*^Multiple answers allowed (*n* = 730); ^**^Response with class of antimicrobials.

### 3.3 Knowledge of veterinarians on AMU and AMR

**Figure 2:**
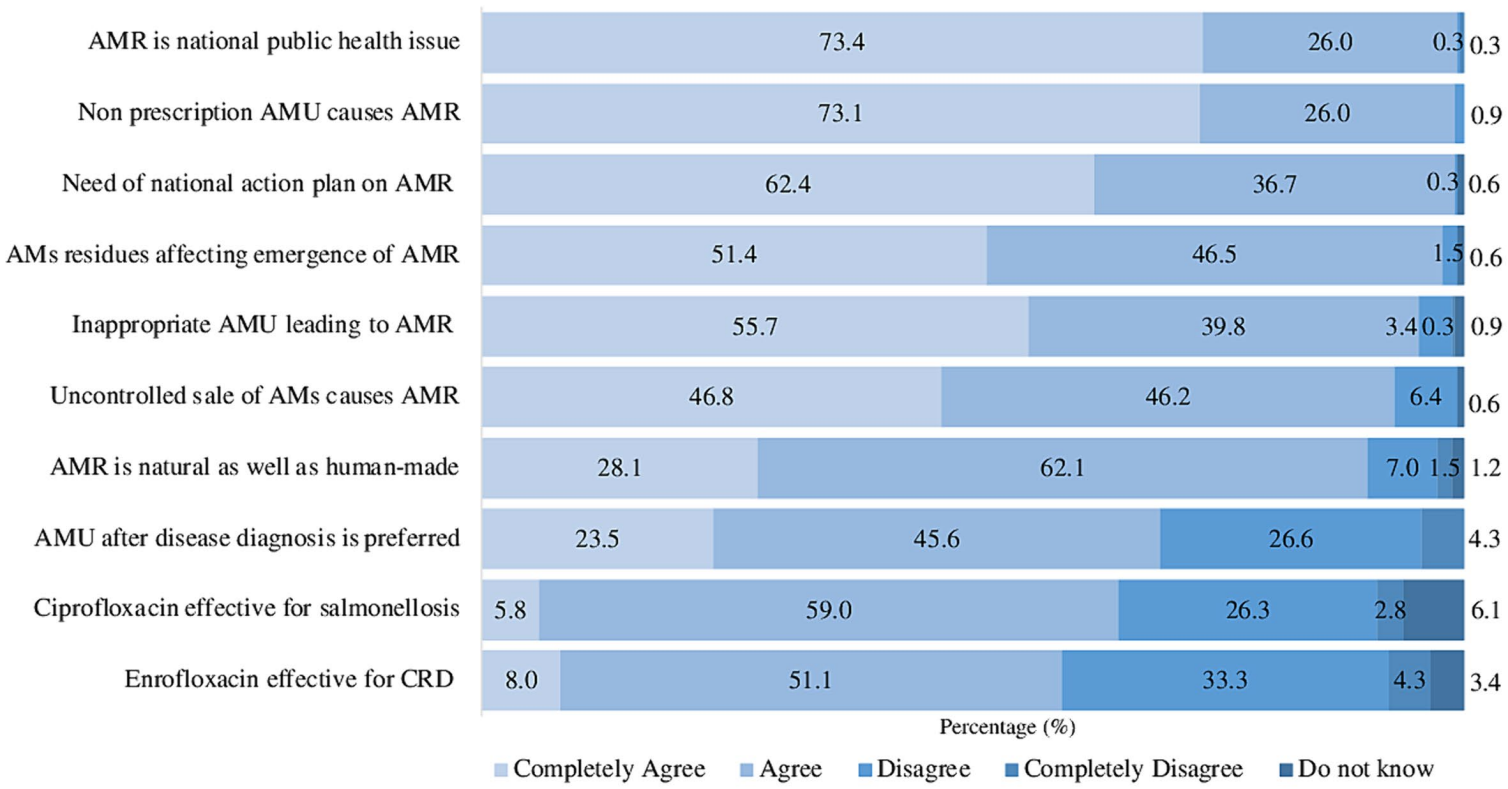
Knowledge of AMU and AMR among veterinarians. AMs, antimicrobials; CRD, chronic respiratory disease.

Ten questions were used to evaluate veterinarians’ knowledge concerning AMU and AMR. A significant majority (59.3%) of respondents provided correct answers, with accurately responding to >80% of the knowledge-related questions, while the remaining 40.7% scored below 80%. The summary responses indicated that they completely agreed and agreed on the following points: (1) AMR is a national public health issue (99.4%), (2) the misuse and overuse of antimicrobials without prescription are the primary factors contributing to AMR (99.1%), and (3) the potential presence of antimicrobial residues leading to the development of AMR (97.9%) (Figure 2). Additionally, 93.0% of veterinarians were aware of the impact of uncontrolled antimicrobial sales in promoting AMR, emphasizing the importance of regulating the distribution and use of antimicrobials. Approximately 69.1% agreed with the statement that it is preferable to use antimicrobials after diagnosing a disease in poultry production. Furthermore, most of them provided correct answers regarding the choice of antimicrobials for treatment (64.8% for salmonellosis and 59.1% for chronic respiratory disease).

### 3.4 Attitudes of veterinarians toward AMU and AMR

**Figure 3:**
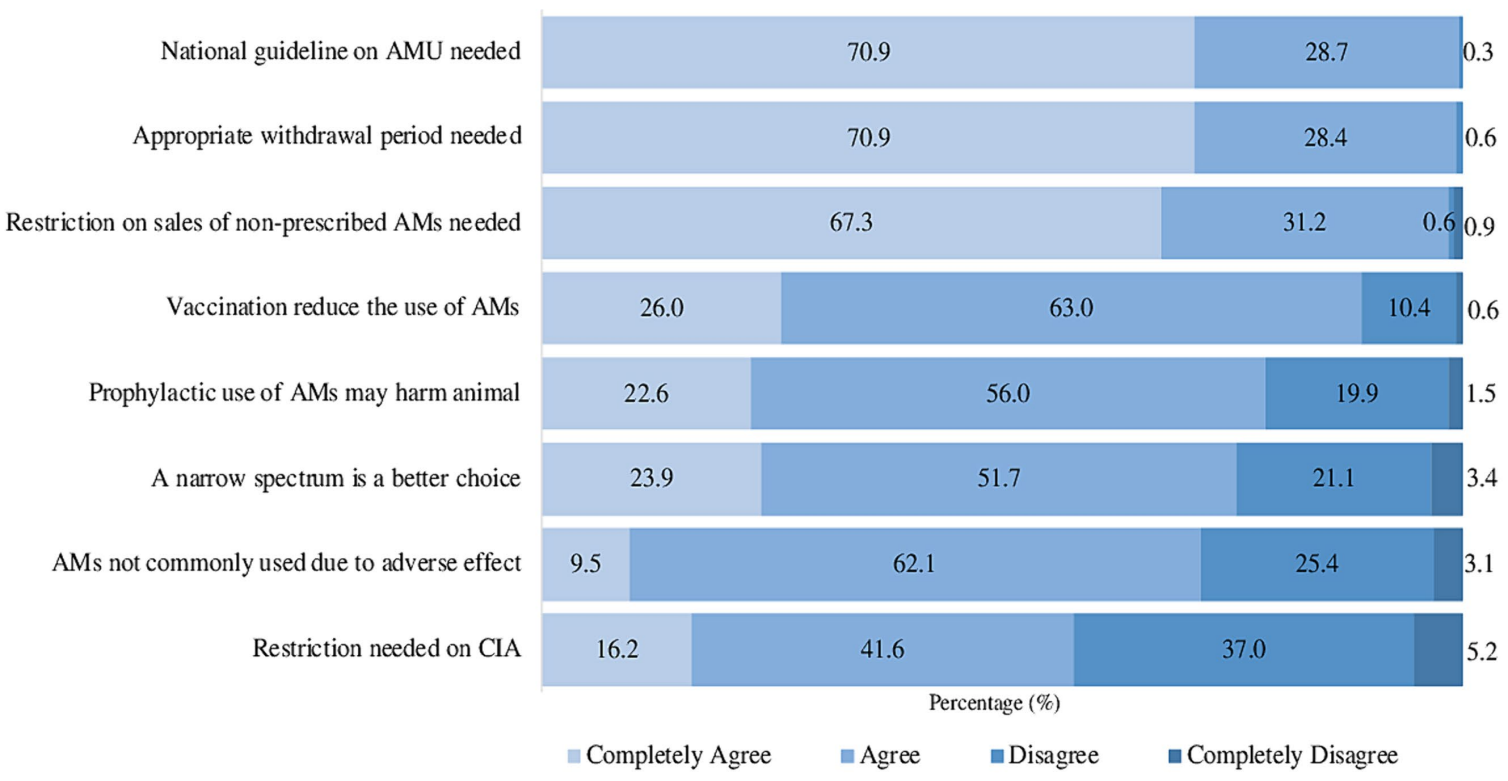
Attitudes toward AMU and AMR among veterinarians. AMs, antimicrobials; CIA, critically important antimicrobial.

A total of 8 questions were designed to assess the attitudes of respondents toward AMU and AMR. A significant proportion (75.3%) of respondents demonstrated proficiency by providing correct answers to over 80% of the questions, while the remaining 24.7% yielded responses falling below 80%. The results indicate that 99.6% agreed that a national guideline on AMU is necessary, and 98.5% supported the idea of prohibiting the sale of non-prescribed antimicrobials. Furthermore, 89.0% of veterinarians believed that vaccination could reduce the use of antimicrobials in poultry production. On the other hand, there was less favorable (78.6%), which addressed that prescribing antimicrobials to healthy animals for disease prevention would harm their poultry health. Three quarters of veterinarians (75.6%) believed that narrow-spectrum antimicrobials were a better choice over broad-spectrum ones. Additionally, 71.6% agreed that antimicrobials are not commonly used in humans and animals due to their adverse effects (Figure 3).

### 3.5 Practices of veterinarians toward AMU and AMR

**Figure 4:**
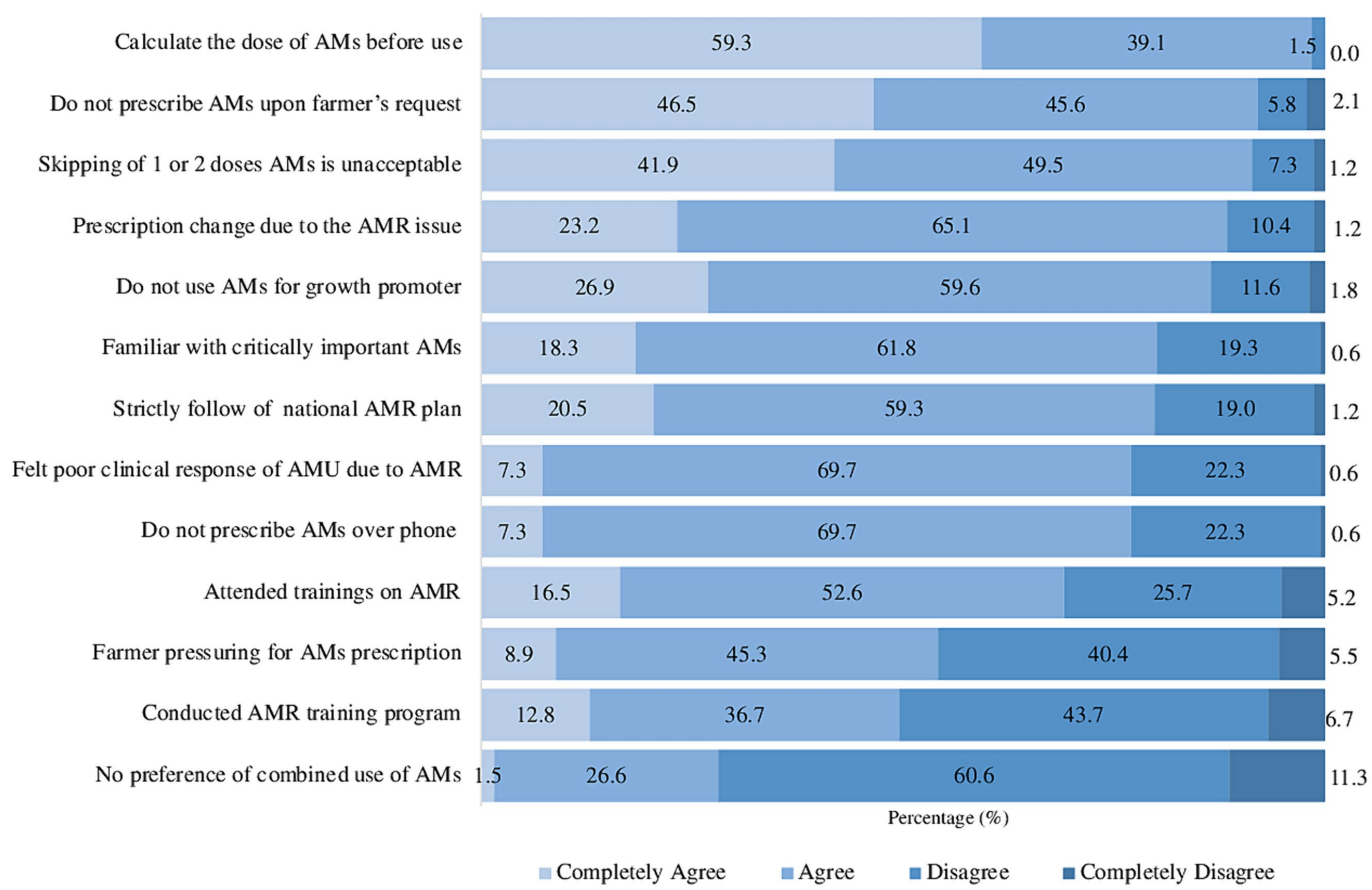
Practices on AMU and AMR among veterinarians. AMs, antimicrobials.

A total of 17 questions were administered to veterinarians regarding their practices in AMU. Among the respondents, 32.5% demonstrated proficiency by correctly answering >80% of the questions, while the remaining 67.5% exhibited responses fell below 80%. Additionally, 88.3% of these veterinarians changed their prescription practices due to the presence of AMR in poultry. This study indicated that 86.5% of poultry veterinarians did not use antimicrobials as growth promoters, and 71.9% did not prefer using combined antimicrobials for therapeutic purposes. Furthermore, 69.1% of poultry veterinarians attended training sessions to update their knowledge about AMU and AMR. Interestingly, approximately 54.2% of poultry veterinarians reported experiencing pressure from farmers to prescribe antimicrobials, while 22.9% prescribed antimicrobials either over the phone or without physically examining the birds (Figure 4).

### 3.6 Factors affecting KAP on AMU and AMR among veterinarians

Demographic data was used to examine its connection with KAP. Regarding knowledge, both univariate and multivariable logistic regression analyses indicated that age serves as a notable predictor (Tables 4, 5). However, no statistical significance was observed among attitudes and practices and demographic factors.

**Table 4:** Univariate logistic regression analysis of demographic factors associated with KAP of veterinarians on AMU and AMR.

Variables		Knowledge			Attitude			Practice		
		Unsat (*N*)	Sat (*N*)	OR (95% CI) *p*-value	Unsat (*N*)	Sat (*N*)	OR (95% CI) *p*-value	Unsat (*N*)	Sat (*N*)	OR (95% CI) *p*-value
Ecozone	Hill	21	129	Ref.	38	112	Ref.	103	47	Ref.
	Mountain	1	11	1.8 (0.2–14.6) 0.6	4	8	0.7 (0.2–2.4) 0.5	8	4	1.1 (0.3–3.8) 0.9
	Terain	23	142	1.0 (0.5–1.9) 0.9	37	128	1.2 (0.7–1.9) 0.5	113	52	1.0 (0.6–1.6) 0.9
Age (year)	24–30	24	157	Ref.	40	141	Ref.	126	55	Ref.
	31–45	15	110	1.1 (0.6–2.3) 0.7	34	91	1.3 (0.7–2.2) 0.3	86	39	0.9 (0.6–1.5) 0.5
	46–60	6	11	0.3 (0.09–0.8) 0.02*	4	13	1.1 (0.3–3.5) 0.9	9	8	0.5 (0.2–1.3) 0.2
	>60		4	Empty	1	3	1.2 (0.1–11.6) 0.9	3	1	1.3 (0.1–12.8) 0.1
Gender	Female	4	45	Ref.	15	34	Ref.	37	12	Ref.
	Male	41	237	0.5 (0.2–1.5) 0.2	64	214	1.4 (0.7–2.8) 0.2	187	91	1.5 (0.7–3.0) 0.2
Province	Bagmati	25	136	Ref.	40	121	Ref.	109	52	Ref.
	Gandaki	4	25	1.1 (0.3–3.6) 0.8	5	24	1.5 (0.6–4.4) 0.4	17	12	1.5 (0.6–3.3) 0.3
	Karnali	2	12	1.1 (0.2–5.2) 0.9	2	12	1.9 (0.4–9.2) 0.3	10	4	0.8 (0.2–2.8) 0.8
	Lumbini	5	49	1.8 (0.6–4.9) 0.2	11	43	1.2 (0.6–2.7) 0.5	36	18	1.0 (0.5–2.1) 0.9
	Madhesh	5	22	0.8 (0.3–2.3) 0.7	10	17	0.6 (0.2–1.3) 0.2	20	7	0.7 (0.3–1.8) 0.5
	Province1	3	27	1.6 (0.5–5.8) 0.4	7	23	1.1 (0.4–2.7) 0.9	22	8	0.8 (0.3–1.8) 0.5
	Sudurpaschim	1	11	2.0 (0.2–16.3) 0.5	4	8	0.7 (0.2–2.3) 0.6	10	2	0.4 (0.1–1.9) 0.3
Education level	Bachelor	25	151	Ref.	44	132	Ref.	119	57	Ref.
	Master	20	123	1.0 (0.5–1.9) 0.9	34	109	1.1 (0.6–1.8) 0.8	99	44	0.9 (0.5–1.5) 0.7
	Ph.D.	0	8	Empty	1	7	2.3 (0.3–19.5) 0.4	6	2	0.7 (0.1–3.5) 0.7
Type of primary service	Academia and research	10	64	Ref.	17	57	Ref.	55	19	Ref.
	Government service	17	78	0.7 (0.3–1.7) 0.4	19	76	1.2 (0.6–2.4) 0.6	60	35	1.6 (0.9–3.2) 0.1
	Non-government organization	3	19	0.9 (0.2–4.0) 0.9	5	17	1.0 (0.3–3.1) 0.9	17	5	0.8 (0.3–2.6) 0.8
	Private business	15	121	1.3 (0.5–3.0) 0.6	38	98	0.8 (0.4–1.5) 0.4	92	44	1.3 (0.3–2.6) 0.3

Unsat, unsatisfactory; Sat, satisfactory; OR, odds ratio; Ref., reference group.

**Table 5:** Multivariable logistic regression analysis of risk factors associated with knowledge of veterinarians on AMU and AMR.

Predictor	Adjusted OR	Std err.	95% CI	*p*-value
Age group (years)				
24–30	Ref.			
31–45	1.12	0.39	0.56–2.23	0.85
46–60	0.28	0.15	0.09–0.83	0.02
>60	–	–	–	–
Constant	6.541	1.43	4.25–10.05	<0.0001

AIC = 261.45. OR, odds ratio; Ref., reference group; Std err., standard error; CI, confidence interval; AIC, Alkaline Information Criteria.

Several variables showed a statistically significant association with the KAP of veterinarians regarding AMU and AMR (Supplementary Table S1). Notably, practitioners who were aware of the harmful consequences associated with inappropriate AMU in animals, leading to AMR in humans, exhibited a knowledge quotient 8.5 times higher than their counterparts lacking this awareness. Moreover, specific aspects within veterinarians’ cognitive framework, such as understanding “the utilization of antimicrobials subsequent to disease diagnosis” (OR = 8.7, *p* < 0.0001), “antimicrobials residues can lead to AMR” (OR = 43.3, *p* = 0.001) and “lack of control in sales of antimicrobials” (OR = 3.1, *p* = 0.02), have emerged as pivotal risk factors influencing their comprehension of AMU.

Significant factors are notably associated with attitudes, including the knowledge of the “adverse effects of antimicrobials” (OR = 22.9, *p* < 0.0001), belief in the statement that “vaccines can mitigate AMU” (OR = 8.6, *p* < 0.0001), and “prophylactic use of antimicrobials may harm healthy animal” (OR = 8.1, *p* = <0.0001). Similarly, specific elements related to practices in antimicrobial stewardship are subject to certain influential risk factors, including belief in inclination for “AMU for growth promotion” (OR = 5.4, *p* = 0.002), inclination toward “prescribing antibiotics upon a farmer’s request” (OR = 6.1, *p* = 0.016), “familiar with WHO’s CIA list” (OR = 9.3, *p* < 0.0001), and responsiveness to “changing antimicrobials due to AMR concerns” (OR = 9.7, *p* = 0.002).

### 3.7 Association among the KAP on AMU and AMR among veterinarians

The finding suggests a significant positive correlation between the level of knowledge regarding AMU and AMR among veterinarians and their corresponding attitudes (Table 6). Specifically, veterinarians who possessed a strong attitude of AMU and AMR demonstrated practices that were 1.7 times more favorable (*p* = 0.01).

**Table 6:** Association among the KAP on AMU and AMR among veterinarians.

Variable	Adjusted OR (95% C.I.)	*p-*value
Knowledge and attitudes	0.7 (0.3–1.5)	0.03
Knowledge and practices	2.1 (0.9–4.5)	0.06
Attitudes and practices	1.7 (1.2–4.0)	0.01

## 4 Discussion

### 4.1 Demographic distribution and AMU situation

Most participants were concentrated in the Terai and Hill regions, with a relatively small number working in the mountain region. Several reasons could explain this distribution, including a higher human population density, increased commercial poultry farming activities, increased availability of academic institutions, and public and private veterinary services in the Terai and Hill regions compared to the mountain region (16, 17). Additionally, the majority of veterinarians were young, belonging to the age group of 24–30 years. This contrasts with a study conducted in Nigeria where 52.8% of veterinarians were in the 30–39 age group (15). This result indicated a greater inclination among young veterinarians for employment in the poultry industry. Of the 327 veterinarians, 51.7% worked in public services such as public service, academia, and research, which was similar to a previous study, where 44.4% of veterinarians worked in public service (15). Only 15.0% of veterinarians were female, which was lower than the overall ratio of female veterinarians among registered veterinarians (22.9%) in Nepal (14). However, a previous study in Bangladesh also reported a similar proportion (14.9%) of female veterinarian engaged in practice (18). This gender distribution discrepancy may be due to the employment of female veterinarians in administrative and academic roles rather than in poultry practices.

### 4.2 AMU situation and farm management

This study revealed that some veterinarians used prohibited antimicrobials in poultry, including day-old chickens. These practices involved combinations of broad-spectrum antimicrobials such as colistin, amoxicillin, gentamicin, tylosin, and tetracycline, with some cases involving more than two antimicrobials. The absence of a veterinary drug act and a designated regulatory authority to oversee the responsible use of antimicrobials in animals may contribute to the occurrence of such malpractices.

Approximately 34.4% agreed that the appropriate treatment guidelines are necessary for an effective strategy to combat AMR. On the contrary, 19.7% of the respondents believed that improving biosecurity and hygiene in farms, and 17.1% believed that increasing educational campaigns could be effective in combating AMR. This finding aligns with recent studies indicating that veterinarians are involved in all aspects of AMR, including prescribing practices, monitoring, and educating farmers (19–22).

### 4.3 KAP among veterinarians on AMU and AMR

Numerous studies conducted in various countries have consistently shown that veterinarians have good KAP related to AMU and AMR, which agreed with the previous study (23–26). Specifically, 86.5% mentioned that they did not use antimicrobials as growth promoters, which was comparable to the finding of Bangladesh (80.0%) (18). Approximately 89.0% of the veterinarians agreed that vaccination could reduce the use of antimicrobials in poultry, and 75.6% believed that using narrow-spectrum antimicrobials are a better choice than broad-spectrum, which is similar with a study conducted in Bhutan (27). Interestingly, most veterinarians (98.5%) expressed that the sale of non-prescribed antimicrobials should be prohibited. Despite this overwhelming agreement, the regulation of antimicrobial prescription in animals in Nepal appears to be weak due to the lack of veterinary drug regulation authority and well-defined legal arrangements.

Half of veterinarians (54.2%) reported experiencing pressure from farmers to prescribe antimicrobials without conducting bird examinations and antimicrobial susceptibility testing. More than 22.9% of veterinarians prescribed antimicrobials over the phone. This finding reflected the farmer’s influence over the veterinarian practices due to the limited availability of veterinarian services in the poultry farming area. The high demand for antimicrobials may be due to their belief that antimicrobials are necessary to maintain the health and productivity of their poultry and a lack of awareness of alternatives to antimicrobials. Consequently, there is a need to expand the coverage of specialist veterinary services both in private and public sectors. Furthermore, 69.1% of veterinarians attended training sessions to update their knowledge about AMU, which was higher than a previous study (47.2%) (15). This difference may be attributed to the availability of various AMR stewardship programs conducted by national and international organizations in Nepal, compared to the previous study conducted in Nigeria (15). However, approximately 16.2% of veterinarians were unfamiliar with the Critically Important Antibiotics (CIA) listed provided by the World Health Organization (WHO). This lack of awareness can potentially lead to the inappropriate use of antimicrobials, especially in the last resort antibiotics such as carbapenems and polymyxin. The misuse of these antibiotics can lead to the emergence of multidrug-resistant bacteria, posing significant challenges to public health as these infections become more challenging to treat. WHO has recognized this as a serious concern and included it in their global priority list of AMR bacteria (28).

### 4.4 Risk factors related to KAP of veterinarians on AMU and AMR

The logistic regression analysis reveals a significant disparity in knowledge regarding AMU and AMR between veterinarians aged 24–30 and those in the 45–60 age group, with an OR of 0.28. This implies that veterinarians aged 45–60 exhibit increased awareness of AMU and AMR, possibly stemming from the recent incorporation of these issues. Veterinarians with knowledge of the adverse consequences of inappropriate AMU (OR = 8.5), AMU following disease diagnosis (OR = 8.7), and concerns about the lack of control in antimicrobial sales contributing to AMR (OR = 3.1) demonstrated significantly higher levels of knowledge. These findings emphasize the need for targeted educational interventions to enhance awareness among veterinarians, addressing key aspects such as the proper use of antimicrobials, timing of AMU, and regulatory oversight. Furthermore, veterinarians endorsing the belief that vaccination can reduce AMU demonstrated attitudes toward AMU and AMR that were 8.6 times more positive than those who did not share this belief. This emphasizes the substantial impact of vaccination perceptions on veterinarians’ attitudes, aligning with the One Health approach (29). The positive correlation suggests that veterinarians acknowledging the potential of vaccines to decrease AMU may be more inclined toward proactive and preventive measures, contributing to responsible antimicrobial practices. This highlights the important of integrating and promoting positive vaccination beliefs in veterinary education, aligning with global efforts to combat AMR (3). Moreover, veterinarians who refrain from using antimicrobials as growth promoters exhibit attitudes 5.4 times more favorable toward AMU and AMR compared to counterparts not adhering to such practices. This underscores the influence of responsible AMU choices on veterinarians’ perceptions, attributed to their high level of knowledge on AMR (30).

These findings highlight specific areas for targeted interventions and emphasize the importance of global guidelines in shaping veterinarian’s behaviors. Overall, the study provides valuable insights for promoting prudent antimicrobial use within veterinary practices. One limitation of this study was the use of an online questionnaire for survey administration as a potential source of non-response bias. To mitigate this potential bias, efforts were made to provide all participants with comprehensive information regarding the objective of this study.

## 5 Conclusion

The study revealed that most veterinarians demonstrated good KAP regarding AMU and AMR. However, trend in AMU practices have been identified, including the prescribing antimicrobials over the phone without physical examination or conducting necessary laboratory tests. There is an urgent need to enhance continuing education and establish regulations to ensure their adherence to the WHO’s list of Clinically Important Antimicrobials for human medicine to restrict their use in poultry. The study also highlights the necessity to expand specialized veterinary services with laboratory testing facilities at the farm level, ensuring an adequate number of veterinarians, and implementing effective antimicrobial stewardship programs in the poultry industry. To counterbalance AMR, the promotion of alternative strategies such as vaccination and enhanced biosecurity measures is recommended. These strategies align with efforts to safeguard public health by reducing the risk of AMR.

## Data availability statement

The original contributions presented in the study are publicly available. This data can be found at: https://figshare.com/s/2afef4a4764f9819f80e.

## Ethics statement

The studies involving humans were approved by Nepal National Health Research Council. The studies were conducted in accordance with the local legislation and institutional requirements. The participants provided their written informed consent to participate in this study.

## Author contributions

MS: Writing – original draft, Formal analysis, Data curation. SJ: Writing – review & editing, Visualization, Validation, Supervision, Funding acquisition, Conceptualization.
