# Supplementary material for: Knowledge, attitudes, and practices on antimicrobial use and antimicrobial resistance among poultry practitioner veterinarians
Source: Front Vet Sci. 2024 Apr 9;11:1349088. doi: 10.3389/fvets.2024.1349088 (PMC11036543; doi:10.3389/fvets.2024.1349088)
Supplement: Supplementary file 1 [file Table_1.DOCX]

Supplementary Material

# Supplementary Data

**Supplementary Table S1**. Univariate logistic regression analysis of KAP among veterinarians regarding AMU and AMR

| Predictors | Score | Response | | OR | 95% CI | *P*-value |
| --- | --- | --- | --- | --- | --- | --- |
|  |  | **Disagree** | **Agree** |  |  |  |
| **Knowledge** |  |  |  |  |  |  |
| The inappropriate use of antimicrobials in animals can contribute to the development of AMR in humans. | Unsatisfied | 8 | 37 | Ref. |  |  |
|  | Satisfied | 7 | 275 | 8.5 | 2.9-24.8 | <0.0001 |
| AMR is a natural as well as human-made phenomenon. | Unsatisfied | 17 | 28 | Ref. |  |  |
|  | Satisfied | 15 | 267 | 10.8 | 4.9-24.0 | <0.0001 |
| Antibiotics are used when animals have been diagnosed with a disease by a veterinarian. | Unsatisfied | 33 | 12 |  |  |  |
|  | Satisfied | 68 | 214 | 8.7 | 4.2-17.7 | <0.0001 |
| Antimicrobial residues can lead to the emergence of AMR. | Unsatisfied | 6 | 39 | Ref. |  |  |
|  | Satisfied | 1 | 281 | 43.3 | 5.1-368.7 | 0.001 |
| A lack of control in the sale of antibiotics contributes to AMR. | Unsatisfied | 7 | 38 | Ref. |  |  |
|  | Satisfied | 16 | 266 | 3.1 | 1.2-7.9 | 0.021 |
| **Attitudes** |  |  |  |  |  |  |
| Antimicrobials are not commonly used in humans and animals due to their adverse effects. | Unsatisfied | 61 | 18 | Ref. |  |  |
|  | Satisfied | 32 | 216 | 22.9 | 12.0-43.5 | <0.0001 |
| Vaccination can reduce the use of antimicrobials in poultry farms. | Unsatisfied | 24 | 55 | Ref. |  |  |
|  | Satisfied | 12 | 236 | 8.6 | 4.0-18.2 | <0.0001 |
| A broad-spectrum antimicrobial is not a better choice, when a narrow spectrum is available. | Unsatisfied | 45 | 34 | Ref |  |  |
|  | Satisfied | 34 | 214 | 8.3 | 4.7-14.8 | <0.0001 |
| Prescribing antimicrobials to healthy animals as a form of prophylaxis may harm the health of the animal. |  | 41 | 38 | Ref. |  |  |
|  |  | 29 | 219 | 8.1 | 4.5-14.7 | <0.0001 |
| **Practices** |  |  |  |  |  |  |
| I do not use antimicrobials as growth promoter. | Unsatisfied | 40 | 184 | Ref. |  |  |
|  | Satisfied | 4 | 99 | 5.4 | 1.9-15.5 | 0.002 |
| I do not prescribe the antibiotics upon farmer’s request | Unsatisfied | 24 | 200 | Ref. |  |  |
|  | Satisfied | 2 | 101 | 6.1 | 1.4-26.2 | 0.016 |
| Farmer does not pressure me for antimicrobials prescription. | Unsatisfied | 121 | 103 | Ref. |  |  |
|  | Satisfied | 29 | 74 | 3.0 | 1.8-5.0 | <0.0001 |
| I adhere strictly to the National Antimicrobial Resistance Plan concerning AMR. | Unsatisfied | 60 | 164 | Ref. |  |  |
|  | Satisfied | 6 | 97 | 5.0 | 2.5-14.2 | <0.0001 |
| Skipping one or two doses during an antimicrobial course is not considered acceptable. | Unsatisfied | 24 | 200 | Ref. |  |  |
|  | Satisfied | 4 | 99 | 3.0 | 1.0-8.8 | 0.049 |
| I have modified my prescription practices in response to the AMR issue in poultry. | Unsatisfied | 36 | 188 | Ref. |  |  |
|  | Satisfied | 2 | 101 | 9.7 | 2.3-41.0 | 0.002 |
| I am familiar with the WHO's critically important list of antimicrobials. | Unsatisfied | 61 | 163 | Ref. |  |  |
|  | Satisfied | 4 | 99 | 9.3 | 3.3-26.3 | <0.0001 |
| I have participated in training sessions and conferences to stay updated on AMU and AMR. | Unsatisfied | 96 | 128 | Ref. |  |  |
|  | Satisfied | 5 | 98 | 14.7 | 5.8-37.5 | <0.0001 |
| Opting for combined antibiotics to ensure therapeutic success is not the preferred approach. | Unsatisfied | 169 | 55 | Ref. |  |  |
|  | Satisfied | 66 | 37 | 1.7 | 1.0-2.9 | 0.035 |
| I have changed an antimicrobial agent because of resistance confirmed on AST. | Unsatisfied | 65 | 159 | Ref. |  |  |
|  | Satisfied | 4 | 99 | 10.1 | 3.6-28.6 | <0.0001 |

Note: OR: odds ratio; Ref: reference group; CI: confidence interval
